# Performance Evaluation and Kinetic Analysis of Photocatalytic Membrane Reactor in Wastewater Treatment

**DOI:** 10.3390/membranes10100276

**Published:** 2020-10-08

**Authors:** Zeyad Zeitoun, Ahmed H. El-Shazly, Shaaban Nosier, Mohamed R. Elmarghany, Mohamed S. Salem, Mahmoud M. Taha

**Affiliations:** 1 Chemical Engineering Department, Faculty of Engineering, Alexandria University, Alexandria 11432, Egypt; zeyad.zeitoun@alexu.edu.eg (Z.Z.); ahmed.elshazly@ejust.edu.eg (A.H.E.-S.); nosier2017@gmail.com (S.N.); 2 Chemical and Petrochemical Engineering Department, Egypt-Japan University of Science and Technology (E-Just), New Borg El-Arab City, Alexandria 21934, Egypt; 3 Mechanical Power Engineering Department, Faculty of Engineering, Mansoura University, Mansoura 35516, Egypt; mohamedsameh@mans.edu.eg; 4 Mansoura University Nanotechnology Center, Mansoura University, Mansoura 35516, Egypt; 5 Environmental Engineering Department, University of Science and Technology, Zewail City of Science and Technology, October Gardens, Giza 12578, Egypt

**Keywords:** photocatalytic membrane reactor, wastewater treatment, titanium dioxide, photocatalysis, membrane distillation, polyvinylidene fluoride (PVDF), membrane characterization

## Abstract

The objectives of the current study are to assess and compare the performance of a developed photocatalytic membrane reactor (PMR) in treating industrial waste (e.g., organic dye waste) against membrane distillation. The current PMR is composed of a feed tank, which is a continuous stirred photocatalytic reactor containing slurry Titanium dioxide (TiO_2_) particles that are activated by using ultraviolet lamp irradiation at a wavelength of 365 nm, and a poly-vinylidene flouride (PVDF) membrane cell. The experimental setup was designed in a flexible way to enable both separate and integrated investigations of the photocatalytic reactor and the membrane, separately and simultaneously. The experimental work was divided into two phases. Firstly, the PVDF membrane was fabricated and characterized to examine its morphology, surface charge, and hydrophobicity by using a scanning electron microscope, surface zeta potential, and contact angle tests, respectively. Secondly, the effects of using different concentrations of the TiO_2_ photocatalyst and feed (e.g., dye concentration) were examined. It is found that the PMR can achieve almost 100% dye removal and pure permeate is obtained at certain conditions. Additionally, a kinetic analysis was performed and revealed that the photocatalytic degradation of dye follows a pseudo-first-order reaction.

## 1. Introduction

The textile industry is one of the largest sources yielding tons of waste dyeing effluents that, even at low concentrations, reduce wastewater transparency, oxygen solubility, and are generally toxic [1,2]. Therefore, significant attention was directed to investigate different approaches for waste treatment before being discharged to the environment to meet the limitations imposed by legislation [3]. Over the years, wastewater treatment methods have advanced beyond conventional methods (i.e., coagulation, filtration, adsorption, etc.) to overcome the complexity and diversity of pollutants existing in domestic, industrial, and agro-industrial waste-streams and to provide clean drinking water to confront the population growth and water scarcity issues.

**Figure 1:**
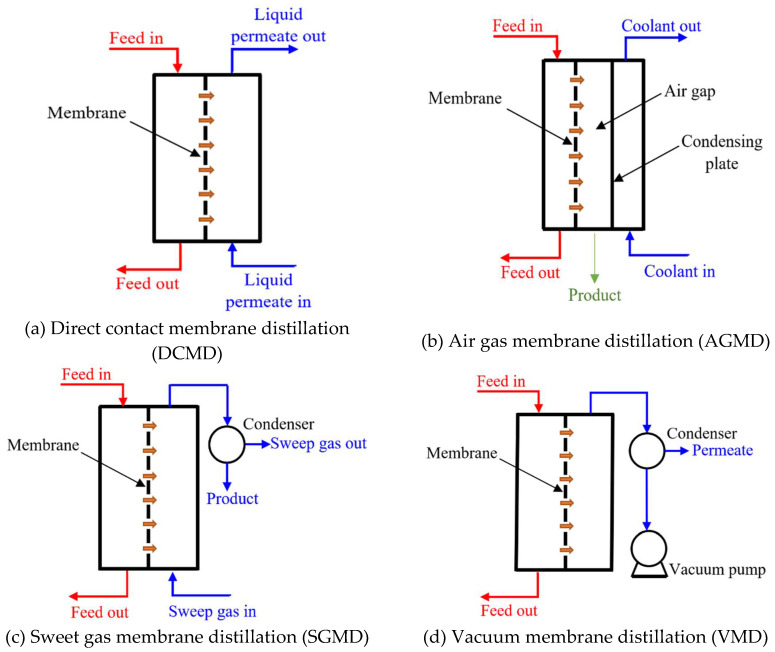
The four membrane distillation configurations.

For instance, thermally-driven membrane processes have shown notable capabilities in removing different contaminants existing in wastewater [4]. Although there are four main configurations used for membrane distillation (MD) [5,6], as shown in Figure 1, the direct contact membrane distillation (DCMD) configuration [7] is the most widely used due to its simplicity and ease of application. Moreover, the performance of different membrane fabrication materials was investigated, and it was found that polyvinylidene fluoride (PVDF), as a material for membrane fabrication, is a promising one due to its good film-forming ability, thermal stability, high mechanical strength, and excellent chemical and aging resistance [8]. However, adopting MD for wastewater management was hindered due to some limitations; such as membrane fouling, wetting, high level of feed pretreatment requirements, and inability of some membranes to remove microcontaminants [9].

Another promising method of wastewater treatment is photocatalysis. In general, photocatalysis has gained considerable attention in wastewater treatment in recent years owing to its ability to completely oxidize and mineralize organic pollutants. Several studies investigated using different photocatalytic substances such as (TiO_2_, ZnO_2_, CeO_2_, ZrO_2_, WO_3_, V_2_O_5_, Fe_2_O_3_, etc.) and sulfides (CdS, ZnS, etc.). A detailed explanation of the mechanism of organic pollutants removal by photocatalysis can be found elsewhere [10,11,12,13,14]. From these studies, it is revealed that, titanium dioxide (TiO_2_) has several advantages over other photocatalysts such as (i) mechanical and chemical stability (meaning that it retains its characteristics and composition with time and at normal operating temperatures), (ii) non-poisonous and non-toxic properties, (iii) the ability to be used under visible light not only the UV-light, (iv) super-hydrophilicity, and (v) commercial availability [15]. However, this method (photocatalysis) also suffers from difficulties, especially in photocatalyst recovery. 

The limitations accompanied by membrane separation processes and photocatalytic degradation of contaminants can be solved by coupling both methods to form a hybrid system named photocatalytic membrane reactors (PMRs). The performance of different PMRs combinations in terms of fabrication materials of membranes and photocatalysts have been assessed in the open literature [14,16,17]. It was found that an integrated system of polyvinylidene fluoride (PVDF) membrane along with ${\mathrm{TiO}}_{2}$ photocatalyst seems to be a promising solution for wastewater treatment owing to the previously mentioned advantages of the PVDF as well as photocatalysis by using ${\mathrm{TiO}}_{2}$. 

The performance of this integrated system (i.e., PVDF and ${\mathrm{TiO}}_{2}$) have been examined under different conditions in previous studies in the open literature as listed in Table 1. As shown in Table 1, several methods were used to fabricate membranes. Although each method has its procedures and product properties [18], the electrospinning technique was adopted in the current study owing to its capabilities of obtaining a highly porous membrane and consequently high fluxes [19]. The PMRs configurations examined in the literature are divided into (i) slurry reactors with photocatalysts suspended in a feed solution and (ii) photocatalyst-supported membranes. Grzechulska et al. [20] compared the performance of both configurations and showed that the first configuration is more efficient than the second one. Thus, this configuration (a slurry reactor with photocatalysts suspended in a feed solution) was also adopted in the current study. 

The objectives of the current study are to design and develop a flexible hybrid PMRs system to investigate the potential use of PMRs for wastewater treatment (i.e., degradation of organic dyes “methylene blue—MB”) and compare its performance against conventional membrane distillation (MD). Additionally, kinetic analysis of photodegradation of MB by ${\mathrm{TiO}}_{2}$ without MD is also carried out.

## 2. Materials and Methods

### 2.1. Membrane Fabrication

**Figure 2:**
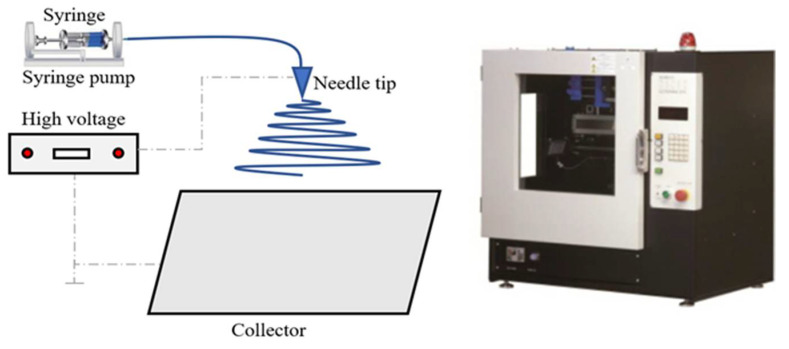
Electrospinning device and mechanism.

Raw PVDF pellets (ρ=1.78 $g/{\mathrm{cm}}^{3}$, average Mw ~275,000 g/mol), purchased from Sigma Aldrich, St. Louis, MO, USA, were allowed to form a homogeneous solution of 16 wt% PVDF and 84 wt% mixture of N-Dimethyl acetamide (DMAc) and acetone (20 vol% DMAc, 80 vol% acetone) after 12 h of continuous magnetic stirring of solution components at 90 °C. Stirring is directly followed by the electrospinning process (shown in Figure 2) to preserve solution homogeneity and not to affect the membrane morphology. The electrospinning voltage is set at 20 kV, the solution was pumped at a flow rate of 1 mm/h, spinneret speed (needle speed) is 100 mm/s, cleaning frequency and interval are 15 min and 1 s, respectively. Spinning distance, the distance traveled by the needle in one direction, is 15 cm. These electrospinning conditions are listed in Table 2; afterward, the prepared membranes were put in an oven at 60 °C for 24 h to remove the solvents residuals. Finally, this fabricated membrane was fully characterized to ensure free-beads fibers and to test its morphology, surface charge, and hydrophobicity. Characterization includes SEM, surface zeta potential, and contact angle measurements.

### 2.2. Membrane Characterization and Analysis

#### 2.2.1. Membrane Morphology

**Figure 3:**
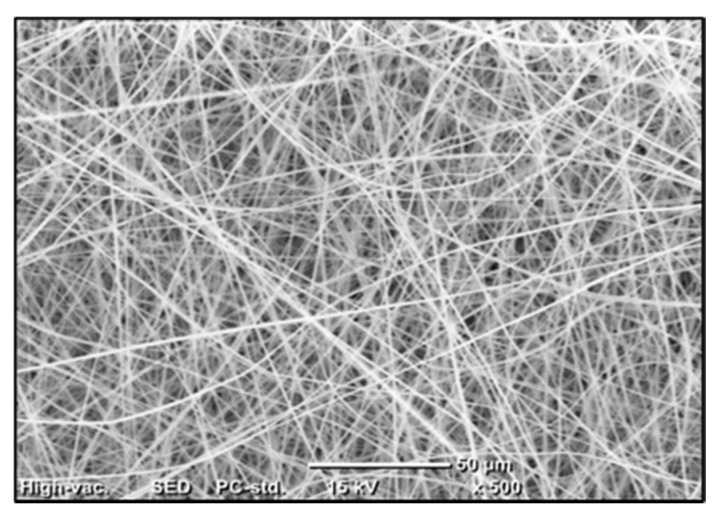
SEM image for PVDF membrane.

Membrane morphology was examined using a Scanning Electron Microscopy “SEM” (JEOL JSM-6010LV), Figure 3 shows a randomly aligned intercrossing fiber network with uniform fiber diameter distribution throughout the structure which reveals the consistency of the electrospinning adopted conditions [34].

#### 2.2.2. Surface Zeta Potential

**Figure 4:**
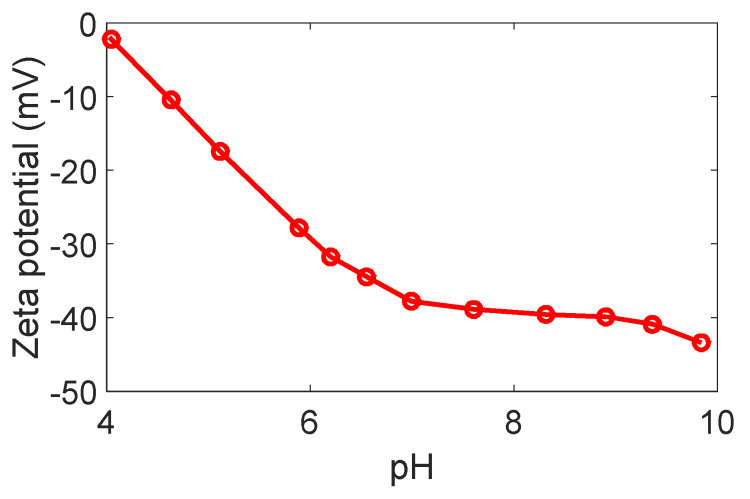
Zeta potential of the PVDF membrane surface.

Streaming current method using an electro-kinetic analyzer (Surpass Anton Paar, Saint Laurent, Austria) was conducted to measure the PVDF membrane surface zeta potential. The membrane sample was cut into 0.2 cm × 0.1 cm pieces and then immobilized on an adjustable gap cell. Determination of zeta potential was carried out by KCl solution (1.0 mmol/L) and other prepared solutions were utilized to study the effect of solution pH on the charge density of the membrane surface. Adjusting the pH value was done by hydrochloric acid (HCl) and potassium hydroxide (KOH). Visolab for Surpass performed the necessary calculations. Figure 4 shows that the membrane’s surface is negatively charged in the range of −5 to −45 mV at pH values from 4 to 10.

#### 2.2.3. Membrane Contact Angle

**Figure 5:**
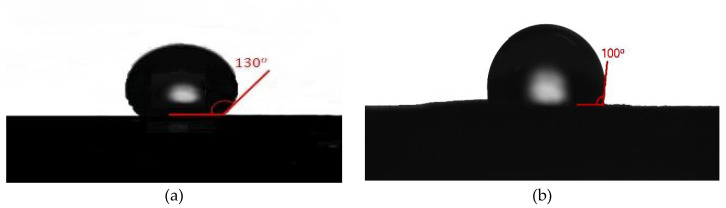
Contact angle: (**a**) water droplet on PVDF membrane surface [36]; (**b**) methylene blue droplet on PVDF membrane surface.

**Figure 6:**
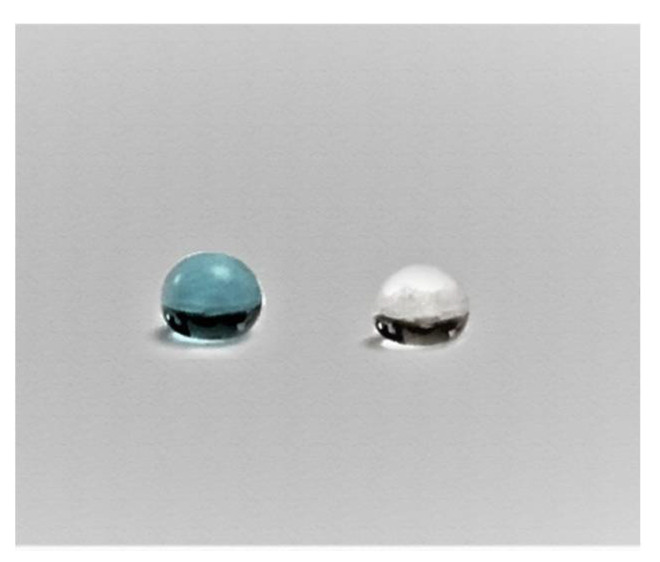
Images of dye and water droplets on PVDF membrane.

Hydrophobicity of the membrane was analyzed by the water contact angle test (Theta Lite, Biolin Scientific, Sweden) [35]. In this test, the contact angle between the membrane sample and methylene blue droplet was measured and compared to the previously measured contact angle between the membrane sample and water droplet [36], as shown in Figure 5 and Figure 6. The contact angle between the PVDF membrane and water drop phase is equal to ($130^{ο}$) while that between methylene blue drop phase and PVDF membrane is equal to ($100^{ο}$). This test proves that the hydrophobicity of the membrane decreased when using methylene blue solution instead of pure water and this will have a significant effect on the purification of water as a consequence of the prevention of dye passage through the membrane [37].

### 2.3. TiO_2_ Characterization and Analysis

**Figure 7:**
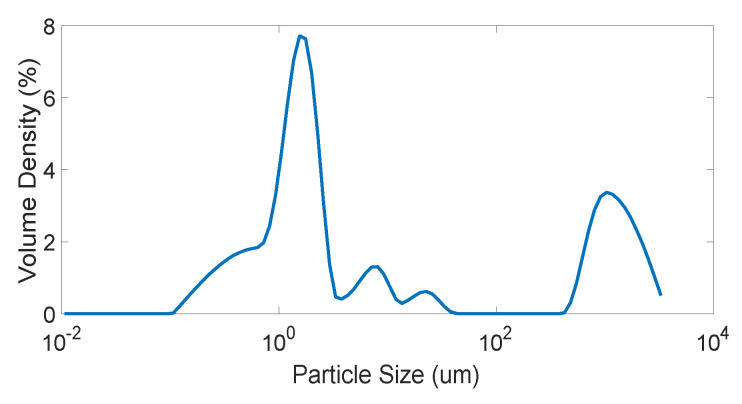
Particle size distribution of ${\mathrm{TiO}}_{2}$.

High quality Anatase (≫99%) ${\mathrm{TiO}}_{2}$ was purchased from Sigma Aldrich, USA. The particle size and phases existing were examined by implementing particle size distribution and XRD analysis, respectively. Particle size distribution was determined by using N5 submicron particle size analyzer, BeckMan Coulter, with using water as the diluent. Analysis of the particle size distribution, shown in Figure 7, shows that the mean particle size lies between 1 and 4 µm with a small number of large particles greater than 400 µm.

**Figure 8:**
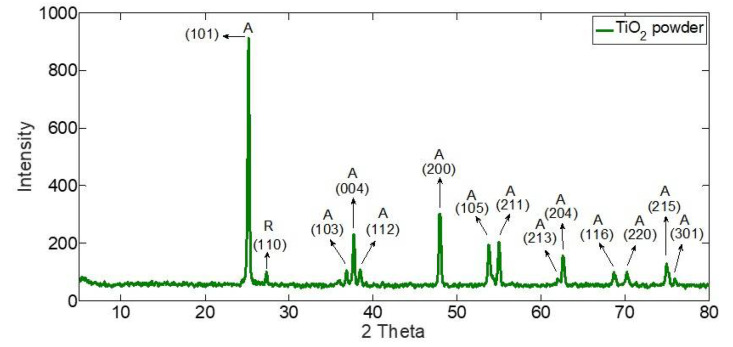
XRD patterns for ${\mathrm{TiO}}_{2}$ nanoparticles.

Figure 8 shows the X-Ray Diffraction (Shimadzu XRD-6100) results. It is revealed that the strongest peaks are at 2θ equals 25.4174˚, 37.9077˚, and 48.1766˚ corresponding to the miller indices (101), (004), and (200), respectively. The values of the detected diffraction angles in this study are quantitively consistent with the findings of previous studies [38,39] as well all other peaks are in good agreement with the standard spectrum (JCPDS no.: 88-1175 and 84-1286) [39]. Therefore, it is confirmed that the ${\mathrm{TiO}}_{2}$ used is mainly in the anatase (A) phase (≫99%) with traces of rutile (R) [40].

## 2.4. Experimental Setup

**Figure 9:**
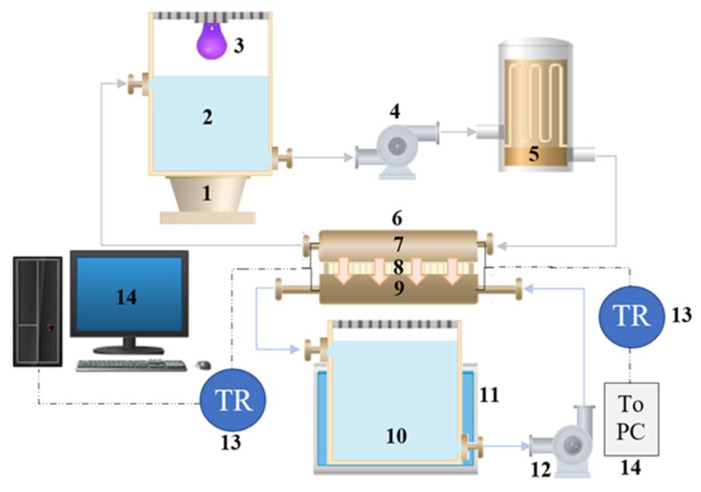
Hybrid system diagram: (1) magnetic stirrer; (2) feed tank; (3)UV-lamp; (4) feed pump; (5) heating unit; (6) membrane cell; (7) feed compartment; (8) PVDF membrane; (9) permeate compartment; (10) permeate tank; (11) cooling jacket; (12) permeate pump; (13) thermocouples; (14) PC data acquisition system.

The photocatalytic membrane reactor (PMR) utilized in the current study, shown in Figure 9, consists of a direct contact membrane distillation (DCMD) cell and a photocatalytic reactor with ${\mathrm{TiO}}_{2}$ nanoparticles as a slurry in the feed tank. The membrane cell consists of two compartments separated by the poly-vinylidene flouride (PVDF) membrane. The temperature difference across the membrane is achieved by introducing cold deionized water into the lower compartment of the membrane where it is combined with condensed vapors passing through the membrane from the upper compartment and is subsequently collected in a permeate tank. The feed tank contains ${\mathrm{TiO}}_{2}$ nanoparticles kept suspended within the dye (i.e., methylene blue) by using a magnetic stirrer. The ${\mathrm{TiO}}_{2}$ nanoparticles are activated by UV-lamp irradiation at a wavelength of 365 nm. Before entering the membrane cell, the feed solution is introduced, by using a micro-pump (Micropump L20561 A-Mount Suction Shoe Pump Head; Cole-Parmer, Vernon Hills, IL, USA), into a copper coil immersed into a water bath heater to raise the feed temperature to 55 °C for membrane distillation. Afterward, the feed solution is fed to the upper compartment of the membrane cell counter-currently to the permeate stream. The concentrate stream, leaving the upper compartment of the membrane cell, is recirculated to the feed tank. It is worthy of mention that, inlet and outlet temperatures of membrane cell streams (i.e., inlet streams are feed and deionized water; outlet streams are the permeate and concentrate) were measured by using thermocouples (K-type, ±2.2 °C or ±0.75%) to keep temperature difference fixed throughout the experiments. The system was designed and constructed in a flexible way to enable both separate and integrated investigations of the membrane and the photocatalytic reactor, separately and simultaneously, to end up with a comparison between the performance of the MD and the integrated PMR. 

## 2.5. Experimental Procedures

Each experiment starts with mixing and dissolving a predetermined amount of dye (i.e., methylene blue “MB”) into distilled water in the stirred feed tank to prepare the desired dye solution concentration. Dye dispersion is maintained by using sonication. Meanwhile, the water bath heater is turned on to increase bath temperature to 70 °C. Preliminary experiments have shown that this bath temperature (70 °C) is sufficient to increase feed solution temperature to 55 °C before entering the DCMD cell. Afterward, the feed pump is turned on to allow the circulation of feed solution through the experimental setup (i.e., feed tank, water bath heater, and DCMD cell). Each experiment lasts for 4 h and 2 mL samples from permeate and concentrate streams were withdrawn every 30 min. The weight and concentration of the permeate were measured and recorded during each sample. Additionally, the feed concentration was determined to analyze the effect of ${\mathrm{TiO}}_{2}$ on MB degradation.

As mentioned earlier, the experimental setup was designed in a flexible way to enable both separate and integrated experiments. Thus, in the current study, the performance of membrane distillation and integrated photocatalytic membrane reactor was evaluated for different dye and ${\mathrm{TiO}}_{2}$ concentrations. The examined dye concentrations range from 4 to 15 ppm while the ${\mathrm{TiO}}_{2}$ concentrations range from 0.0 to 0.3 g/L.

## 2.6. Data Representation and Analysis

### Membrane Flux and Dye Removal Efficiency

Changes in permeate weight were monitored and the permeate flux (J) was defined as the mass of water passed through the membrane per unit time under fixed temperature and pressure [41]; it is calculated from the following Equation (1) [42].

(1) $$J=\frac{m}{A\cdot t}$$

where J is the permeate flux ($\mathrm{kg}/(m^{2}\cdot \mathrm{hr})$), m is the permeate mass (kg), A is the effective membrane area ($m^{2}$) and t is the sampling time (h). Feed and permeate concentrations were followed by using a UV-Visible Spectrophotometer (Hitachi U-3900). The optimum wavelength was 664 nm which is in the visible light range. The dye removal efficiency was calculated at the end of each cycle from Equation (2):

(2) $$\eta \;=\left(1-\frac{C_{p}}{C_{f}}\right)\;\cdot \;100\%$$

where η is the dye removal efficiency, $C_{p}$ and $C_{f}$ are permeate and initial feed concentration, respectively.

## 3. Results and Discussion

### 3.1. Membrane Distillation

**Figure 10:**
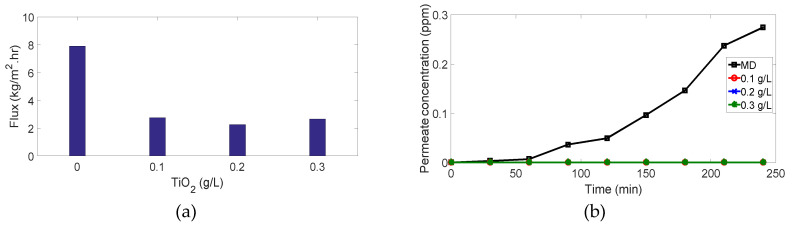
Results for 4 ppm methylene blue (MB) experiments: (**a**) flux vs. amount of ${\mathrm{TiO}}_{2};$ (**b**) permeate concentration vs. time.

**Figure 11:**
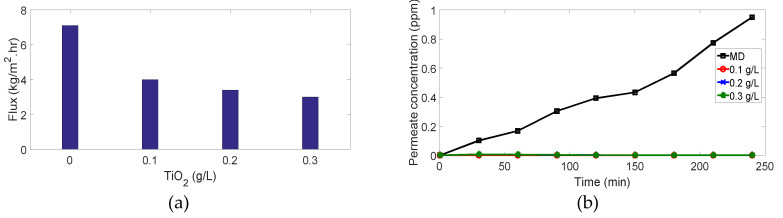
Results for 7 ppm MB experiments: (**a**) flux vs. amount of ${\mathrm{TiO}}_{2};$ (**b**) permeate concentration vs. time.

**Figure 12:**
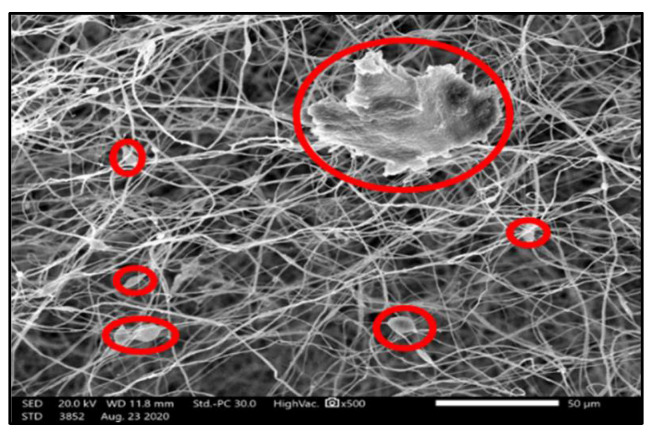
SEM for PVDF membrane after dye treatment.

As shown in Figure 10 and Figure 11, membrane distillation experiments (without photocatalytic reactor) for 4 ppm and 7 ppm methylene blue recorded the highest flux and lowest dye removal (91.1% and 87.84%, respectively) in comparison with other experiments using the same MB concentration. This is attributed to the high adsorption of the dye onto the membrane as the MB dissociates in aqueous solutions into a cation (the chromophore, ${\mathrm{dye}}^{+}$) and an anion (${\mathrm{Cl}}^{-}$). Accordingly, there is an attractive force between the ${\mathrm{dye}}^{+}$ and the negatively charged membrane leading to the ease of MB adsorption on the membrane and passage through the pores, as shown in Figure 12. Moreover, high membrane porosity, as measured in our previous study [36], and lower hydrophobicity as proved by measuring the contact angle will result in low removal and high flux.

**Figure 13:**
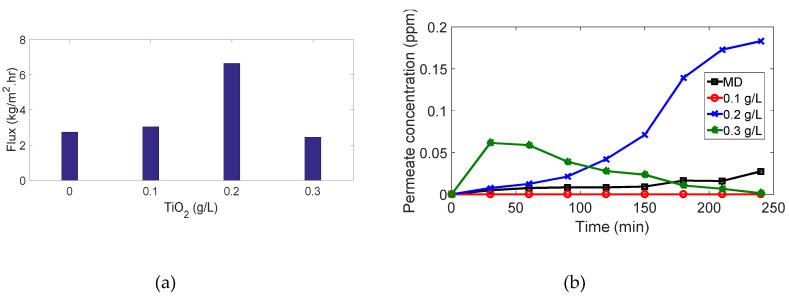
Results for 11 ppm MB experiments: (**a**) flux vs. amount of ${\mathrm{TiO}}_{2};$ (**b**) permeate concentration vs. time.

**Figure 14:**
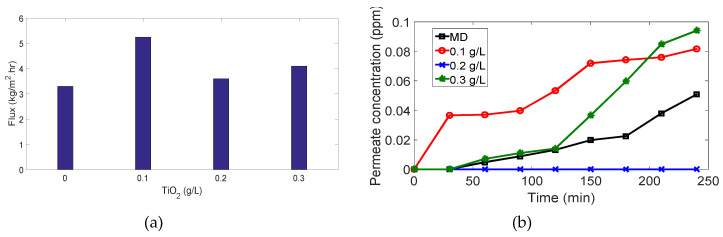
Results for 15 ppm MB experiments: (**a**) flux vs. amount of ${\mathrm{TiO}}_{2};$ (**b**) permeate concentration vs. time.

Membrane distillation for 11 ppm and 15 ppm did not achieve the highest flux as 4 ppm and 7 ppm in comparison to other experiments with the same MB concentration. Hence, the drop in flux, as shown in (Figure 13a and Figure 14a), results from the high organic foulant “MB” loading which induces concentration polarization owing to the retention and partially blocking of the membrane pores. Membrane fouling by dye molecules is a complex phenomenon that is still not well understood. It is thought that this phenomenon mainly results from the interactions between the dye molecules and membrane. These interactions can be represented as physicochemical interactions, i.e., hydrophobic interactions (dispersion forces), polar interactions (dipole forces), and charge transfer (hydrogen bonding). These interactions significantly affect the permeate flux and the percentage of dye removal. Moreover, partial pore wetting may cause a reduction in the permeate flux. It is worth mentioning that an illustration of various forms of pore wetting can be found elsewhere [43].

Although the permeate flux decreased, the percentage of dye removal was remarkably high: it reached 99.7% and 99.6% for 11 ppm and 15 ppm, respectively, (Figure 13b and Figure 14b). Although deposition of the dye on the membrane surface occurs due to the attraction force between the negatively charged membrane and dye ions as mentioned above, retention of molecules increases gradually during the process and covers the membrane surface leading to repulsion between dye molecules adsorbed on the surface and dye molecules in the feed solution. This phenomenon is named (dye-dye fouling) and leads to the prevention of dye molecules from passing through the membrane, therefore, removal efficiency increases.

### 3.2. PMR Performance

For MB concentration of 4 and 7 ppm, (Figure 10 and Figure 11): interestingly, when we added the photocatalytic reactors with different ${\mathrm{TiO}}_{2}$ concentrations to the above experiments, 100% removal was achieved but with lower fluxes. The enhancement of dye removal is due to the presence of ${\mathrm{TiO}}_{2}$ which degrades MB molecules under UV irradiation by photocatalysis mechanism. Fluxes are decreased as the ${\mathrm{TiO}}_{2}$ may enter the membrane cell and block the pores and thus prevent vapors passage through the membranes.

For MB concentration of 11 ppm, Figure 13: meanwhile, 100% and 98.1% dye removal were recorded in case of 0.1 g/L and 0.2 g/L ${\mathrm{TiO}}_{2}$ concentrations, respectively. There is an important behavior was observed in case of 0.3 g/L ${\mathrm{TiO}}_{2}$ concentration as shown in Figure 13. At first, the permeate concentration and flux increase gradually followed by a gradual decrease in concentration and flux. This may be explained as follows; (i) During the first 30 mins, ${\mathrm{TiO}}_{2}$ nanoparticles were not activated yet therefore its effect was not obvious leading to a high load of the MB on the membrane surface as a result of attraction forces as explained above as well the MB molecules were able to penetrate the membrane. (ii) After 30 mins, ${\mathrm{TiO}}_{2}$ was activated and dye-dye fouling occurred so the flux and concentration decreased remarkably. 

For MB concentration of 15 ppm, Figure 14: in this case, different behavior of the system’s performance was observed. Degradation of MB occurred, and this decreased the high load of organic molecules on the membrane leading to the increase in permeate flux. The removal efficiency was recorded to be 99.4%, 100%, and 99.3% when ${\mathrm{TiO}}_{2}$ concentration changed from 0.1 g/L, 0.2 g/L, and 0.3 g/L respectively. As shown in Figure 14, an integrated system of a photocatalytic reactor containing 0.2 g/L ${\mathrm{TiO}}_{2}$ as slurry and membrane cell serving as a distillation unit is the optimum design under these conditions. Table 3 summarizes the performance of MD and PMR performance.

### 3.3. Feed Concentration Analysis

**Figure 15:**
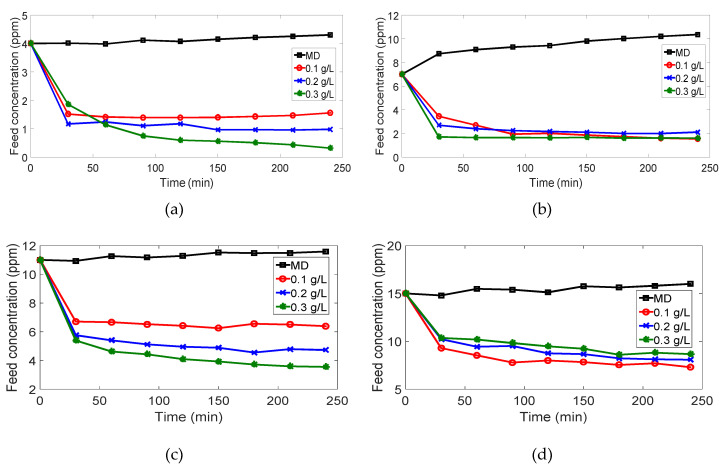
Feed concentration vs. time: (**a**) 4 ppm; (**b**) 7 ppm; (**c**) 11 ppm; (**d**) 15 ppm.

As mentioned earlier, the change in feed concentration was recorded and presented in Figure 15 to analyze the effect of ${\mathrm{TiO}}_{2}$ on MB degradation. There are two noticeable opposing effects; (i) an increase in feed concentration is detected throughout the MD experiments while (ii) a reduction in the feed concentration is observed in the case of PMR experiments. The feed concentration increase is attributed to the water vapor passage through the PVDF membrane. On the other hand, the feed concentration reduction is mainly due to the photodegradation of MB by ${\mathrm{TiO}}_{2}$ in the feed tank.

### 3.4. Photocatalysis Kinetic Analysis 

Kinetics analysis of MB degradation was carried out in a batch reactor under the optimum condition of the PMR system, listed in Table 4. The reaction was performed in a glass reactor containing 500 mL of MB solution and the respective amount of photocatalyst. At first, the solution was continuously stirred by a magnetic stirrer at room temperature in the absence of light for 60 mins to ensure that equilibrium adsorption on the surface of the photocatalyst has been reached; then, 2 mL samples were withdrawn every 30 s. Afterward, the samples were purified from any existing traces of ${\mathrm{TiO}}_{2}$ by using centrifugation at 6000 rpm for 20 mins. Then, the concentrations of these purified samples were measured by UV-Spectrophotometer at a wavelength of 664 nm. 

The concentration-time data obtained were used to determine the reaction order by fitting the data to the linear relationship of pseudo-first-order (Lagergren’s rate law; Equation (3)) and the pseudo-second-order rate law Equation (4).

(3) $$\ln\;\left(q_{e}-q_{t}\right)=\ln\left(q_{e}\right)-K_{1}t$$

(4) $$\frac{t}{q_{t}}=\frac{1}{K_{2}q_{e}^{2}}+\;\frac{t}{q_{e}}$$

where $q_{t}$ and $q_{e}$ are the adsorption capacities  (mg/g); amount adsorbed of MB per unit mass of ${\mathrm{TiO}}_{2}$, at time t and equilibrium, respectively. $K_{1}$ and $K_{2}$ represent pseudo-first-order rate constant ($\min^{-1}$) and pseudo-second-order rate constant (g/(mg·min)), respectively. 

**Figure 16:**
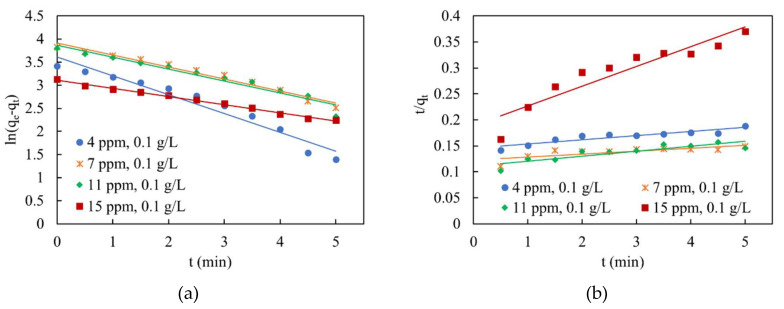
Kinetic data fitting: (**a**) pseudo-first-order kinetic model fitting; (**b**) pseudo-second-order kinetic model fitting.

As shown in Figure 16, it was found that the photodegradation of MB was described as a first-order reaction because the correlation coefficient $\left(R^{2}\right)$ for the pseudo-first-order model is higher than that of the pseudo-second-order model, additionally, the theoretical $q_{e}$ is more consistent with the calculated $q_{\mathrm{cal}}$ for the pseudo-first-order model. For each of the fittings, the reaction rate constants, theoretical $q_{e}$, calculated $q_{\mathrm{cal}}$ and the correlation coefficient $\left(R^{2}\right)$ were determined and summarized in Table 5 [44].

### 3.5. Comparison between PMRs and Previous Studies

**Figure 17:**
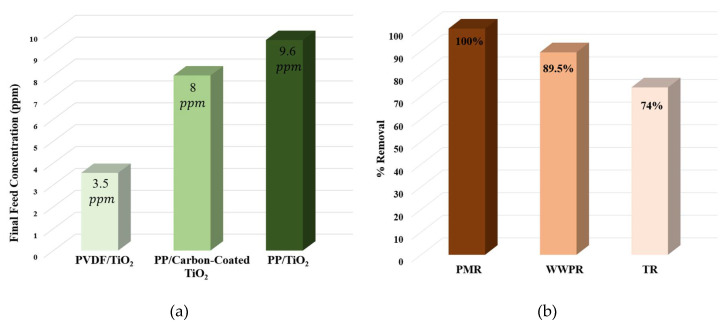
Comparison between: (**a**) PVDF/TiO_2_, polypropylene (PP)/carbon-coated TiO_2_, and PP/TiO_2_ systems; (**b**) PMR, wetted wall photocatalytic reactor (WWPR), and tubular reactor (TR).

The performance of the developed PMR system was compared against different PMR systems [45] operating under the conditions of initial MB concentration 11 ppm, photocatalyst load 0.1 g/L, and operating time 4 hrs. As shown in Figure 17a, the current PMR system achieves the least final feed concentration compared to different PMR system of polypropylene (PP) membrane combined with either a slurry of ${\mathrm{TiO}}_{2}$ or carbon-coated ${\mathrm{TiO}}_{2}$ which emphasizes the capabilities of the current system. 

Additionally, the performance of the current PMR in treating MB was compared against the wetted wall photocatalytic reactor (WWPR) [46], and tubular reactor (TR) [47] at different operating conditions (see Table 6). As shown in Figure 17b, 100% MB removal was achieved by utilizing the developed PMR system at an initial MB concentration of 11 ppm and ${\mathrm{TiO}}_{2}$ load of 0.1 g/L. Meanwhile, 89.5% and 74% were recorded in case of the WWPR and the TR, respectively. It is worth mentioning that the PMR is privileged by attaining pure permeate, for further usage, as well as achieving high MB photocatalytic degradation.

## 4. Remarks and Conclusions

The key findings of the current study are as follows:

- Electrospinning conditions adopted in the current study enable the acquisition of free beads fibers with a negatively charged surface and a high hydrophobicity membrane.
- The performance of the PMR exceeds the conventional MD, thus allowing more water to be reused which is an important advantage from the economic and environmental points of view.
- Using the MD is preferable with high MB concentrations (i.e., 11 and 15 ppm) to obtain nearly pure permeate besides recovering dyes from the concentrate.
- Almost 100% separation efficiency was achieved by operating the PMR at the investigated optimum conditions, which provides high-quality water and low-dyeing waste concentration suitable for discharge.
- Photodegradation of MB on ${\mathrm{TiO}}_{2}$ behaves similarly to the pseudo-first-order kinetic model.

## Figures and Tables

**Table 1:** Summary of previous studies utilizing polyvinylidene fluoride (PVDF)-titanium dioxide (${\mathrm{TiO}}_{2})$ photocatalytic membrane reactors (PMRs).

Membrane		Additives		Pollutant		Ref.
Type	Fabrication Method	Type	Conc.	Type	Conc.	
PVDF	Phase immersion	$\mathrm{DMAc}\_\mathrm{SDS}-\mathrm{GO}/{\mathrm{TiO}}_{2}$	(DMAc_SDS−GO: ${\mathrm{TiO}}_{2}$) 79 wt%:1 wt%	MB	10 ppm	[21]
PVDF/PMMA	Phase inversion	${\mathrm{TiO}}_{2}$	0.12 wt%0.25 wt%0.5 wt%	MB	10 µmol/L	[22]
PVDF-TrFE	Solvent casting	${\mathrm{TiO}}_{2}$	8 wt%	MB	2 ppm	[23]
PVDF-PVP	Electrospinning	${\mathrm{TiO}}_{2}$	4 wt%	MB	3.2 ppm6.4 ppm	[24]
PVDF	Electrospinning	${\mathrm{TiO}}_{2}$	3 wt%6 wt%9 wt%	MB	1 mol/L	[25]
PVDF	Coextrusion	${\mathrm{TiO}}_{2}$	10 wt%20 wt%30 wt%40 wt%	MB	10 ppm	[26]
PVDF	Coextrusion	TiO_2_/MWCNTs	TiO_2_/MWCNTs 10 wt% TiO_2_/MWCNTs 20 wt% TiO_2_/MWCNTs 30 wt% TiO_2_/MWCNTs 40 wt%	MB	10 ppm	[26]
PVDF	Phase inversion	${\mathrm{TiO}}_{2}$	0.5 wt%	MB	10µmol/L	[27]
PVDF	Purchased	${\mathrm{TiO}}_{2}:\mathrm{ZnO}$	(TiO_2_:ZnO) 1:1 (TiO_2_:ZnO) 1:3 (TiO2:ZnO) 1:5	MB	$10^{-5}\;\mathrm{mol}/L$	[28]
PVDF	Phase inversion	$\mathrm{Ag}/{\mathrm{TiO}}_{2}/\mathrm{APTES}$	0.1 g 0.2 g 0.5 g	MB	3 ppm	[29]
PVDF	Dip coating	${\mathrm{TiO}}_{2}$	1 mg/L	MB	10 µM	[30]
PVDF	Dip coating	Titanium isopropoxide	$2\;\times \;10^{-3}$ M	MB	10 µM	[31]
PVDF	Nonsolvent induced phase separation (NIPS)-immersion precipitation inversion	$\mathrm{Ag}/{\mathrm{TiO}}_{2}$	(${\mathrm{TiO}}_{2}:\mathrm{Ag}$) 3.5 wt%:0 wt% (${\mathrm{TiO}}_{2}:\mathrm{Ag}$) 1.57 wt%:17.19 wt% (${\mathrm{TiO}}_{2}:\mathrm{Ag}$) 1.76 wt%:20.54 wt% (${\mathrm{TiO}}_{2}:\mathrm{Ag}$) 1.71 wt%:21.33 wt%	MB	10 mg/L	[32]
PVDF	Phase inversion	${\mathrm{TiO}}_{2}$	0 vol% 6 vol% 12 vol% 21 vol%	MB	0.01 mmo/L	[33]

**Table 2:** Electrospinning condition for preparation of PVDF membrane.

Electrospinning Voltage	20 kV
Flow rate	1 mm/h
Spinneret speed	100 mm/s
Cleaning frequency	15 mins
Cleaning interval	1 s
Spinning distance	15 cm

**Table 3:** Summary—PMR and membrane distillation (MD) performance.

MB Concentration (ppm)	**Percentage of Dye Removal**			
	$\mathrm{Concentration}\;\mathrm{of}\;\mathrm{Ti}O_{2}$ (g/L)			
	0 (MD)	0.1 (PMR)	0.2 (PMR)	0.3 (PMR)
	**After 1 h**			
4	99.82%	100%	100%	100%
7	97.51%	100%	100%	100%
11	99.93%	100%	99.88%	99.45%
15	99.96%	99.74%	100%	99.95%
	**After 2 h**			
4	98.68%	100%	100%	100%
7	93.97%	100%	100%	100%
11	99.92%	100%	99.59%	99.74%
15	99.9%	99.62%	100%	99.9%
	**After 3 h**			
4	95.86%	100%	100%	100%
7	91.03%	100%	100%	100%
11	99.84%	100%	98.6%	99.9%
15	99.84%	99.46%	100%	99.5%
	**After 4 h**			
4	92%	100%	100%	100%
7	84.52%	100%	100%	100%
11	99.7%	100%	98.1%	100%
15	99.6%	99.4%	100%	99.3%

**Table 4:** Optimum conditions for PMR performance.

MB Concentration (ppm)	$\mathrm{Ti}O_{2}\;\mathrm{Concentration}$ (g/L)
4	0.1
7	0.1
11	0.1
15	0.2

**Table 5:** Kinetics parameters for adsorption of MB on ${\mathrm{TiO}}_{2}$ at the optimum conditions.

Optimum Conditions		Rate Law							
MB (ppm)	$\mathrm{Ti}O_{2}$ (g/L)	Pseudo-First-Order Kinetic Model				Pseudo-Second-Order Kinetic Model			
		$q_{e}$ $\left(\frac{\mathrm{mg}}{g}\right)$	$K_{1}$ $\left(\mathrm{mi}n^{-1}\right)$	$q_{\mathrm{cal}}$ $\left(\frac{\mathrm{mg}}{g}\right)$	$R^{2}$	$q_{e}$ $\left(\frac{\mathrm{mg}}{g}\right)$	$K_{2}$ $\left(\frac{g}{\mathrm{mg}\cdot \min}\right)$	$q_{\mathrm{cal}}$ $\left(\frac{\mathrm{mg}}{g}\right)$	$R^{2}$
4	0.1	30.54	0.4069	36.92	0.9487	30.54	$4.39\times 10^{-4}$	125	0.8408
7	0.1	45.87	0.2598	49.97	0.9738	45.87	$2.55\times 10^{-4}$	178.57	0.6164
11	0.1	44.56	0.2587	47.84	0.9489	44.56	$8.3\times 10^{-4}$	104.17	0.7735
15	0.2	22.92	0.1776	22.46	0.9934	22.92	$7.7\times 10^{-3}$	26.25	0.8774

**Table 6:** The effect of PMR, WWPR, and TR on MB removal at different operating conditions.

Operating Conditions	Type of Photocatalytic Reactor		
	PMR	WWPR	TR
Photocatalyst	${\mathrm{TiO}}_{2}$	${\mathrm{TiO}}_{2}/{\mathrm{SiO}}_{2}$	${\mathrm{TiO}}_{2}$
Photocatalyst loading (g/L)	0.1	1.25	0.3
Initial MB concentration (ppm)	11	80	60
Operating time (hrs)	4	2	1
% Removal	100	89.5	74

## Nomenclature

**Symbols**	
A	Membrane area ($m^{2}$)
$C_{f}$	Initial feed concentration (ppm)
$C_{p}$	Permeate concentration (ppm)
J	Permeate flux ($\mathrm{kg}/(m^{2}\cdot \mathrm{hr})$)
$K_{1}$	Pseudo-first-order rate constant ($\min^{-1}$)
$K_{2}$	Pseudo-second-order rate constant (g/(mg.min))
Mw	Molecular weight (g/mol)
m	Permeate mass (kg)
$q_{c}$	Calculated adsorption capacity (mg/g)
$q_{e}$	Equilibrium adsorption capacity (mg/g)
$q_{t}$	Adsorption capacity at time t (mg/g)
$R^{2}$	Correlation coefficient
t	Sampling time (hr)
**Greek letters**	
η	Dye removal efficiency (%)
**Abbreviations**	
Ag	Silver
AGMD	Air Gap Membrane distillation
APTES	Aminopropyltriethoxysilane
CdS	Cadmium Sulfide
${\mathrm{CeO}}_{2}$	Cerium Dioxide
DCMD	Direct Contact Membrane Distillation
DMAc	N-Dimethyl Acetamide
DMAc−SDS−GO	Dimethyl Acetamide-Sodium Dodecyl Sulphate-Graphene Oxide
${\mathrm{Fe}}_{2}O_{3}$	Ferric Oxide
FTIR	Fourier Transform Infrared Spectrometer
KCl	Potassium Chloride
KOH	Potassium Hydroxide
MB	Methylene Blue
MD	Membrane Distillation
MWCNTs	Multi-Walled Carbon Nanotubes
NIPS	Non-solvent Induced Phase Separation
PMMA	Poly-methyl Methacrylate
PMR	Photocatalytic Membrane Reactors
PVDF	Poly-Vinylidene Flouride
PVP	Poly-Vinyl Propylene
SEM	Scanning Electron Microscope
SGMD	Sweep Gas Membrane Distillation
${\mathrm{TiO}}_{2}$	Titanium Dioxide
TrFE	Trifluoro Ethylene
UV	Ultraviolet
VMD	Vacuum Membrane Distillation
$V_{2}O_{5}$	Vanadium Oxide
${\mathrm{WO}}_{3}$	Tungsten Trioxide
XRD	X-Ray Diffraction
ZnO	Zinc Oxide
ZnS	Zinc Sulfide
${\mathrm{ZrO}}_{2}$	Zirconium Dioxide
